# Spectrofluorimetric Determination of Famotidine in Pharmaceutical Preparations and Biological Fluids through Ternary Complex Formation with Some Lanthanide Ions: Application to Stability Studies

**Published:** 2009-06

**Authors:** M. I. Walash, A. El-Brashy, N. El-Enany, M. E. K. Wahba

**Affiliations:** *Department of Analytical Chemistry, Faculty of Pharmacy, Mansoura University, Mansoura, Egypt*

**Keywords:** spectrofluorimetry, lanthanide ions, famotidine

## Abstract

A simple, sensitive and specific method was developed for the determination of famotidine (FMT) in pharmaceutical preparations and biological fluids. The proposed method is based on ternary complex formation of famotidine (FMT) with EDTA and terbium chloride TbCl_3_ in acetate buffer of pH 4. Alternatively, the complex is formed via the reaction with hexamine and either lanthanum chloride LaCl_3_, or cerous chloride CeCl_3_ in borate buffer of pH6.2 and 7.2 respectively. In all cases, the relative fluorescence intensity of the formed complexes was measured at 580 nm after excitation at 290 nm. The fluorescence intensity - concentration plots were rectilinear over the concentration range of 10-100, 5-70, and 5-60 ng/ml, with minimum quantification limits (LOQ) of 2.4, 2.2, and 5.2 ng/ml, and minimum limits of detection (LOD) of 0.79, 0.74, and 1.7 ng/ml upon using TbCl_3_, LaCl_3_, and CeCl_3_ respectively. The proposed method was applied successfully for the analysis of famotidine in dosage forms and in human plasma. The kinetics of both alkaline and oxidative induced degradation of the drug was studied using the proposed method. The apparent first order rate constant and half life time were calculated. A proposal of the reaction pathways is presented.

## INTRODUCTION

**Figure 1:**
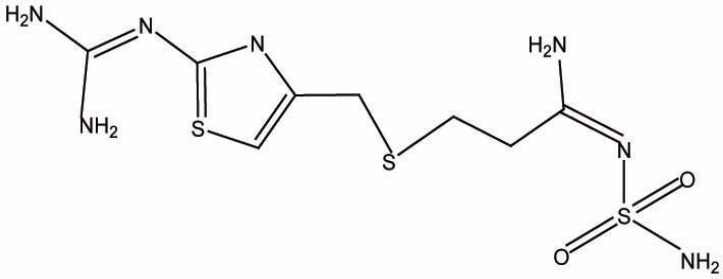
Structural formula of famotidine.

Famotidine (FMT), 3-[2-(diaminomethyleneamino] thiazol-4-ylmethylthio]-N-sulphamoyl propionamidine (Fig. 1) is a histamine H_2_ antagonist which is used in the management of benign gastric and duodenal ulceration, gastro-esophageal reflux, heart burn, and Zollinger-Ellison syndrome (1).

Literature survey reveals many methods for the determination of famotidine in pharmaceutical preparations and biological fluids including: spectrophotometry (2-9), polarography (10), HPLC (11-17), and fluorimetry (18, 19).

The British Pharmacopoeia (BP) (20) recommends a non aqueous potentiometric method for the determination of FMT using perchloric acid as a titrant. The United States Pharmacopoeia (USP) (21) recommends a similar approach for the determination of FMT in its bulk form, and an HPLC for its determination in tablets using a mobile phase consisting of acetate buffer: acetonitrile (93:7) of pH6 with UV detection at 275 nm.

Literature review reveals many methods for the spectrophotometric determination of famotidine, these include the following.

Famotidine has been determined through charge transfer reaction with picrolonic acid at 362 nm (2), ninhydrin at 590 nm (3), tetracyanoquinodimethane at 840 nm (7), and with chloranilic acid at 525 nm (8).

Two methods were developed for the determination of FMT. The first method was based on the reaction between FMT and Fe^3+^- 1,10 phenanthroline and measuring the formed complex at 510 nm. The second method was based on the reaction of the liberated Fe^2+^ with 2, 2-bipyridyl and measuring the product at 520 nm (5).

A reported method for the analysis of Famotidine depends on its reaction with 1, 4 Benzoquinone reagent at pH5.2 (18). The absorbance of the resulting condensation product was measured at 502 nm. The absorbance-concentration plots were rectilinear over the range 40-160 μg.ml^-1^ Furthermore the resulting condensation products exhibited fluorescence at 665 nm after excitation at 290 nm and the calibration graph was rectilinear from 0.4-1.4 μg.ml^-1^.

Another spectrofluorimetric method depends on the synchronous spectrofluorimetric assay of the drug in the presence of fluconazole and ketoconazole using methanol as a solvent (19). The fluorescence intensity of FMT was recorded at 384 after excitation at 284 nm. The calibration curve was linear over the concentration range 15-50 μg/ml.

The proposed method is superior to the previous methods in being more sensitive, simple, less time consuming and having a lower detection limit (LOD; 2.1, 2.2, and 5.2 ng/ml using Tb^+3^, La^+3^, and Ce^+3^ respectively).

Lanthanides have been frequently used for the determination of several compounds of pharmaceutical interest. Terbium for example was utilized for the assay of some catecholamines (22), niflumic acid (23), nalidixic acid (24), heparin (25-26), oxolinic acid (27), and several fluoroquinolone antibiotics such as levofloxacin (28) grepafloxacin (29) pazufloxacin (30) ofloxacin (31) lomefloxacin (32) ciprofloxacin (33) and enoxacin (34).

The proposed method is simple, sensitive and rapid; moreover, it is readily applicable for the determination of FMT in spiked and real human plasma.

## EXPERIMENTAL

### Apparatus

The fluorescence spectra and measurements were recorded using a Perkin Elmer LS 45 Luminescence Spectrometer equipped with a 150 W Xenon discharge lamp and a 1 cm quartz cell.

### Materials and reagents

All reagents and solvents were of Analytical Reagent grade.

1. Famotidine (FMT) was kindly provided by Memphis Chemical Company, Cairo, Egypt. Its purity was checked according to BP (20) and was found to be 98.8%.
2. Pharmaceutical preparations:
  - Antodine® ampoules (Batch # 22298), labeled to contain 20 mg famotidine/ampoule, and Antodine® tablets (Batch # 3728), labeled to contain 20 mg famotidine/tablet, both are products of Amoun Pharmaceutical Company, Cairo, Egypt;
  - Famotin® tablets (Batch # 384178), labeled to contain 40 mg famotidine/tablet, Memphis Chemical Company, Cairo, Egypt;
  - Servipep® tablets (Batch # 050), labeled to contain 40 mg famotidine/tablet, Novartis Pharma S.A.E, Cairo-C.C.R.111108 under license from Sandoz GmbH, Kundl-Austria;
  - Peptec® tablets (Batch # 044), labeled to contain 20 mg famotidine/tablet, manufactured by Julphar Agency, Egypt.
3. Terbium (III) chloride (Aldrich Chem. Co), 8 × 10^-4^ M aqueous solution was freshly prepared;
4. Lanthanum (III) chloride (Panreac Chemicals), 5 × 10^-4^ M aqueous solution was freshly prepared;
5. Cerous (III) chloride (Fluka), 1 × 10^-3^ M aqueous solution was freshly prepared;
6. Ethylenediamine tetra-acetic acid (di-sodium salt) EDTA, 1.5 × 10^-3^ M aqueous solution was prepared;
7. Hexamine, 10% aqueous solution was prepared;
8. Acetate buffer (pH4.0) was prepared by mixing 0.2 M acetic acid and 0.2 M sodium acetate (20). Borate buffers (pH6.2 and 7.2) were prepared by mixing 0.2 M boric acid with 0.2 M sodium hydroxide (20). The pH has to be checked periodically;
9. Methanol (BDH, Poole,UK).
10. Standard solutions: stock solution was prepared by dissolving 100.0 mg of FMT in 100 ml of methanol and was further diluted with the same solvent as appropriate. The working standard solutions were stable for 7 days when kept in refrigerator.

### General Procedures

**Figure 2:**
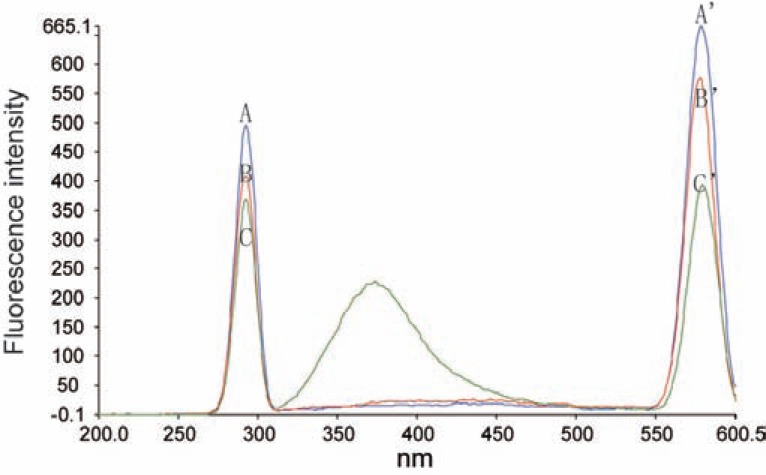
Fluorescence spectra of the formed complex where: B and B' are the excitation and emission spectra respectively of FMT (40 ng/ml) - Tb ^+3^ complex at pH4; A and A' are the excitation and emission spectra respectively of FMT (40 ng/ml) - La ^+3^ complex at pH6.2; C and C' are the excitation and emission spectra respectively of FMT (40 ng/ml)- Ce ^+3^ complex at pH7.2.

**Procedure I (using TbCl_3_).** Aliquot volumes of FMT standard solution covering the working concentration range of 10-100 ng/ml were transferred into a series of 10 ml volumetric flasks. Two ml of TbCl_3_ (8 × 10^-4^ M) were added, followed by 1.0 ml of 1.5 × 10^-3^ M EDTA, and 2 ml of acetate buffer (pH4) and completed to the mark with distilled water ; the fluorescence intensity of the resulting solution was measured at 580 nm after excitation at of 290 nm (Fig. 2). A blank experiment was prepared simultaneously for each measurement. The corrected relative fluorescence intensity was plotted against the final concentration of the drug (ng/ml) to get the calibration curve. Alternatively, the corresponding regression equation was derived.

**Procedure II (using LaCl_3_).** Aliquot volumes of FMT standard solution covering the working concentration range of 5-70 ng/ml were transferred into a series of 10 ml volumetric flasks. Two ml of LaCl_3_(5 × 10^-4^ M) were added, followed by 2 ml of 10% hexamine, and 2 ml of borate buffer (pH6.2) and completed to the mark with distilled water. The procedures as described under “Procedure I” were completed.

**Procedure III (using CeCl_3_).** Aliquot volumes of FMT standard solution covering the working concentration range of 5-60 ng/ml were transferred into a series of 10 ml volumetric flasks. Two ml of CeCl_3_ (1 × 10^-3^ M) were added, followed by 2 ml of 10% hexamine, and 3.5 ml of borate buffer (pH7.2) and completed to the mark with distilled water. The procedures as described under “Procedure I” were completed.

**Procedure for tablets.** Twenty tablets were weighed and pulverized. A weighed quantity of the powder equivalent to 100.0 mg of famotidine was transferred into a small conical flask, and extracted three successive times each with 30 ml of methanol. The extract was filtered into a 100 ml volumetric flask. The conical flask was washed with a few mls of methanol and completed to the mark with the same solvent. Aliquots covering the working concentration range were transferred into 10 ml volumetric flasks. Procedure I, II, or III was applied. The nominal content of the tablets was determined either from the calibration graphs or using the corresponding regression equations.

**Procedure for ampoules.** The contents of 10 ampoules were mixed, aliquots containing 100 mg of FMT were transferred into 100 ml volumetric flask and serial dilution was done with methanol to obtain the working concentration range. Procedure I, II, or III was applied and the nominal content of the ampoule was determined either from the calibration curve or using the corresponding regression equations.

**Procedure for preparation of degradation products.** For kinetic studies, aliquot volumes of FMT standard solution containing 400 μg/ml were transferred into a series of 25 ml volumetric flask to obtain a final concentration of 40 μg/ml. 0.5 M sodium hydroxide, or hydrogen peroxide (1%) were added to prepare the alkaline, or oxidative degradation product respectively. The solution was left in a boiling water bath for 80 minutes in case of alkaline degradation, and at ambient temperature for oxidative degradation. Aliquot volumes of the hydrolyzed solution were transferred to a series of 10 ml volumetric flasks for a fixed time interval (10 minutes), neutralized with 0.5 M hydrochloric acid for alkaline degradation and Procedure I, II, or III was applied. The relative fluorescence intensity of the resulting hydrolyzed solutions after complexation with any of the studied metals was recorded at 580 nm after excitation at 290 nm. log a/a-x *versus* time (minutes) was plotted to get the reaction rate constant and the half life time. Complete degradation was attained by following the same procedure using 2M sodium hydroxide or 30% hydrogen peroxide for alkaline or oxidative degradation. The solution was boiled for 1 hour in case of alkaline degradation, and neutralized with 2 M hydrochloric acid. For oxidative degradation, the solution was allowed to stand for 60 minutes at room temperature, and the excess hydrogen peroxide was expelled by boiling in a water bath for 30 minutes.

**Procedures for spiked human plasma.** Aliquots of human plasma (1.0 ml) were transferred into a series of centrifugation tubes, and spiked with increasing quantities of FMT to get a final concentration range of 20-60 ng/ml. Three ml of 1.2 mM trichloroacetic acid (1 × 3) were added, centrifugation at 3000 rpm for 30 minutes was performed, the aqueous layer was transferred quantitatively to 10 ml volumetric flasks, and Procedure I, II, or III was applied. The nominal content of FMT was determined from the regression equation.

**Procedures for real human plasma.** Servipep® tablet (40 mg famotidine/tablet) was administered to a healthy male volunteer (40 years old). After overnight fasting, 5 mls blood sample was withdrawn 3 hours after administration of the drug, 5 ml of citrate buffer were added, then centrifuged to get about 2-3 ml of plasma. Steps as described under “procedures for spiked human plasma” were followed.

## RESULTS

### Optimization of reaction conditions

The spectrofluorimetric properties of the formed fluorophores as well as the different experimental parameters affecting development and stability of the complex were carefully studied and optimized. Such factors were changed individually while the others were kept constant. These factors include: different types of buffers, pH, volume of the buffer, concentration and volume of the metal ion used, volume of EDTA and hexamine, temperature, and effect of different diluting solvents.

### Effect of addition order

The effect of addition order on the fluorescence intensity of the system was studied. The results show that the addition order of FMT- lanthanide ion-EDTA- acetate buffer or hexamine- borate buffer is the best regarding the fluorescence intensity readings.

### Effect of buffer type and pH

Different types of buffers such as phosphate, citrate, or BRb (Britton Robinson buffer) of the same pH values were studied. It was found that acetate and borate buffers were superior to the other studied buffers since they produced higher fluorescence readings.

**Figure 3:**
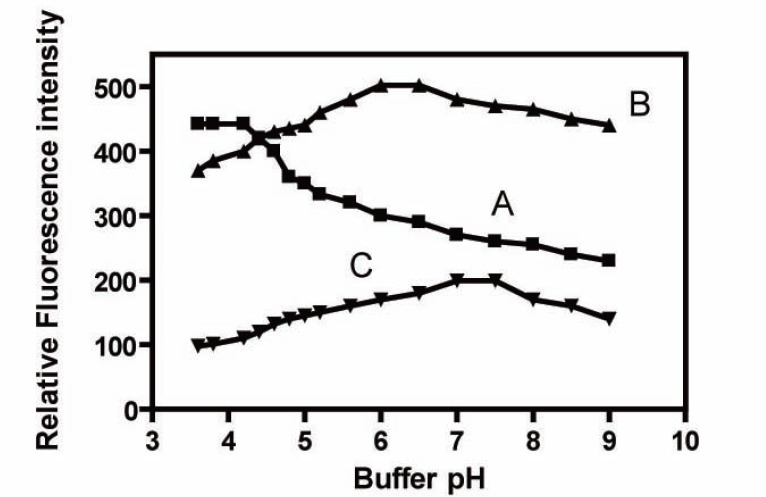
Effect of pH on the fluorescence intensity of (A) FMT-Tb^+3^; (B) FMT-La^+3^; and (C) FMT-Ce^+3^ complexes (FMT=40 ng/ml).

The influence of pH of the buffer on the fluorescence intensity of the formed complexes was investigated over the pH range 3.5–5.6 (acetate buffer) and from 6-8.5 (borate buffers). Maximum and constant fluorescence intensities were achieved using acetate buffer pH (3.8-4.2), borate buffer (6-6.5) and (7-7.5) for Tb^+3^, La^+3^, and Ce^+3^ respectively (Fig. 3). Therefore, acetate buffer of pH4 ± 0.2, borate buffer of pH6.2 ± 0.2, and borate buffer of pH7.2 ± 0.2 were used as the optimum pH values throughout the study.

### Effect of Tb^+3^, La^+3^, and Ce^+3^

Keeping all other variables constant, it was found that increasing the volume of Tb^+3^ (8.0 × 10^-4^ M), La^+3^ (5.0 × 10^-4^ M), and Ce^+3^ (1.0 × 10^-3^ M) respectively resulted in a gradual increase in the relative fluorescence of the complexes up to 1.5 ml, after which it remained constant, therefore, 2.0 ± 0.5 ml of the metals was chosen for this study.

### Effect of EDTA and hexamine

Keeping all other variables constant, it was found that increasing the volume of EDTA (1.5 × 10^-3^ M) resulted in a gradual increase in the relative fluorescence intensity of the complexes up to 0.6 ml, after which it remained constant, therefore, 1.0 ± 0.4 ml of the reagent was chosen for this study.

Hexamine was used for La^+3^ and Ce^+3^ since it gives higher fluorescence readings than EDTA. Increasing the volume of hexamine resulted in a subsequent increase in the relative fluorescence values of the formed complexes up to 1.0 ml in case of La^+3^, and up to 1.5 ml in case of Ce^+3^, after which the fluorescence remained constant, therefore 2.0 ± 0.5 ml of 10% hexamine was chosen throughout this study.

### Effect of different diluting solvents

For all the studied lanthanides, different diluting solvents were tested to choose the most suitable one for the complex formation. The investigated solvents include: water, acetonitrile, methanol, dioxane, dimethylsulfoxide, acetone, 0.1 M sodium hydroxide, and 0.1 M hydrochloric acid. The highest fluorescence intensity was obtained using water which adds another advantage to the proposed method. The results are mentioned in Table 1.

**Table 1:** Effect of different diluting solvents on the relative fluorescence intensity of the formed complex (FMT=40 ng/ml)

Solvent	Relative fluorescence intensity		
	TbCl_3_	LaCl_3_	CeCl_3_

Water	442	502	199
Methanol	252	360	165
Acetonitrile	95	50	100
Dimethylsulfoxide	140	192	133
Dimethylformamide	230	175	62
Acetone	10	30	5
0.1 N Sodium hydroxide	350	320	60
0.1 N Hydrochloric acid	240	297	45

The proposed method was applied for the determination of FMT in pure form. The linearity range, detection and quantitation limits (LOD & LOQ) according to ICH Q2B recommendations (37), and performance data using statistical analysis of the results (38), are abridged in Table 2.

**Table 2:** Performance data, detection and quantitation limits (LOD & LOQ) of the proposed method

Parameter	Metal ion		
	TbCl_3_	LaCl_3_	CeCl_3_

Concentration range (ng/ml)	10–100	5–70	5–60
LOD (ng/ml)	0.79	0.74	1.71
LOQ (ng/ml)	2.41	2.24	5.17
Correlation coefficient (r)	0.9999	0.9998	0.9997
Slope	8.90	12.60	4.90
Intercept	2.34	5.36	1.90
Standard deviation of the residuals, S_y/x_	2.97	4.19	8.54
Standard deviation of the intercept of the regression line, S_a_	2.14	2.82	2.56
Standard deviation of the slope of the regression line, S_b_	0.03	0.07	0.07
% Error(RSD%/√n)	0.29	0.33	0.28
%RSD	0.65	0.74	0.63

### Robustness of the method

The robustness of the procedures adopted was demonstrated by the consistency of the relative fluorescence values with the deliberately minor changes in the experimental parameters such as, acetate buffer of pH4 ± 0.2, borate buffer of pH6.2 ± 0.2, and borate buffer pH7.2 ± 0.2 produces a constant relative fluorescence using Tb^+3^, La^+3^, and Ce^+3^ respectively. Changing the volume of the lanthanide ion (2.0 ± 0.5 ml) for Tb^+3^ (8.0 × 10^-4^ M), La^+3^(5.0 × 10^-4^ M), and Ce^+3^ (1.0 × 10^-3^ M) did not greatly affect the relative fluorescence intensity of the formed complex.

Formation constant K_f_ of the reaction product was calculated according to the following equation:

${\text{K}}_{\text{f}}=\frac{\text{F}/{\text{F}}_{\text{m}}}{[{\left(1-\text{F}/{\text{F}}_{\text{m}}\right)}^{\text{n}+1}]{\text{c}}^{\text{n}}{\text{n}}^{\text{n}}}$

where F and F_m_ are the observed maximum fluorescence and the fluorescence obtained from the extrapolation of the two lines obtained from Job’s continuous variation method, respectively; n, is the mole fraction of the reagent (the ratio is 2:1 in case of Tb^+3^ therefore, n=0.3, 1:1 for La^+3^ and Ce^+3^, so n=0.5); C, is the molar concentration of the drug used in Job’s continuous variation method.

Using the above equation, K_f_ was found to be 7.68 × 10^2^, 10.64 × 10^2^ and 6.1 × 10^2^ using Tb^+3^, La^+3^ and Ce^+3^ respectively.

Also, Gibbs free energy changes (G) were calculated according to the following equation (39):

$\Delta \text{G}=-2.303\;\text{RT}\;\text{log}\;{\text{K}}_{\text{f}}$

Where R = gas constant = 8.314 Joule.degree^-1^.mole^-1^; T = absolute temperature = °C + 273

Using the above equation, ΔG was found to be -1.6 × 10^4^, -1.7 × 10^4^ and -1.6 × 10^4^ Joule/Mole applying Tb^+3^, La^+3^ and Ce^+3^ respectively.

The negative value of ΔG indicates that the reaction is spontaneous.

### Mechanism of the reaction

**Figure 4:**
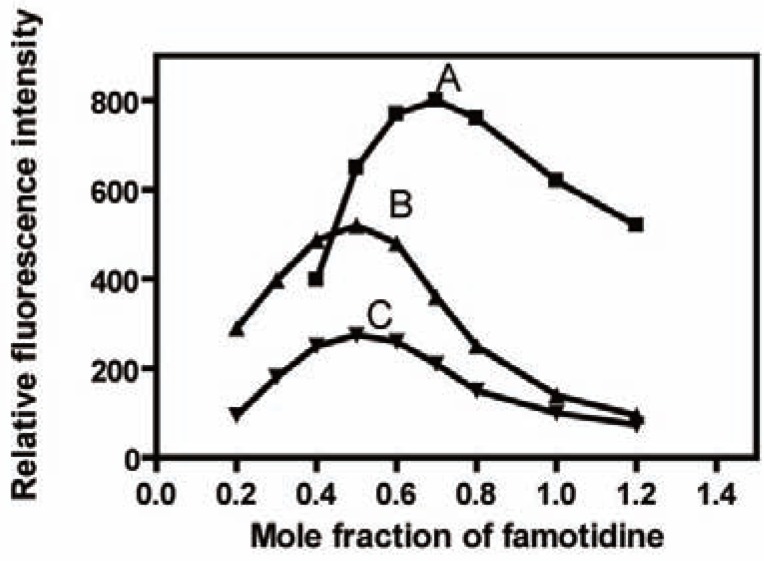
Job’s continuous variation method for: A, FMT with Tb^+3^ (8 × 10^-4^ M for both); B, FMT with La^+3^ (5 × 10^-4^ M for both); C, FMT with Ce^+3^ (1 × 10^-3^ M for both).

**Figure 5:**
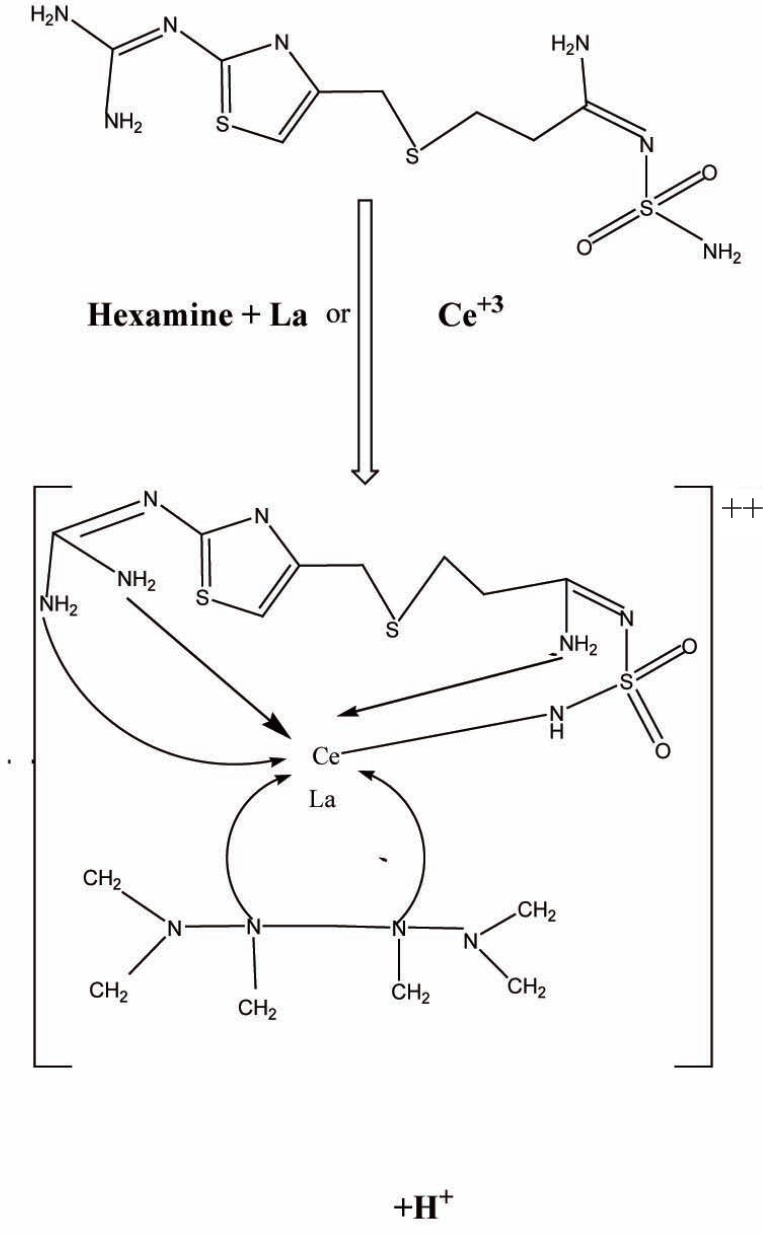
Proposal of the reaction pathway between famotidine and Ce^+3^ or La^+3^ in presence of hexamine.

**Figure 6:**
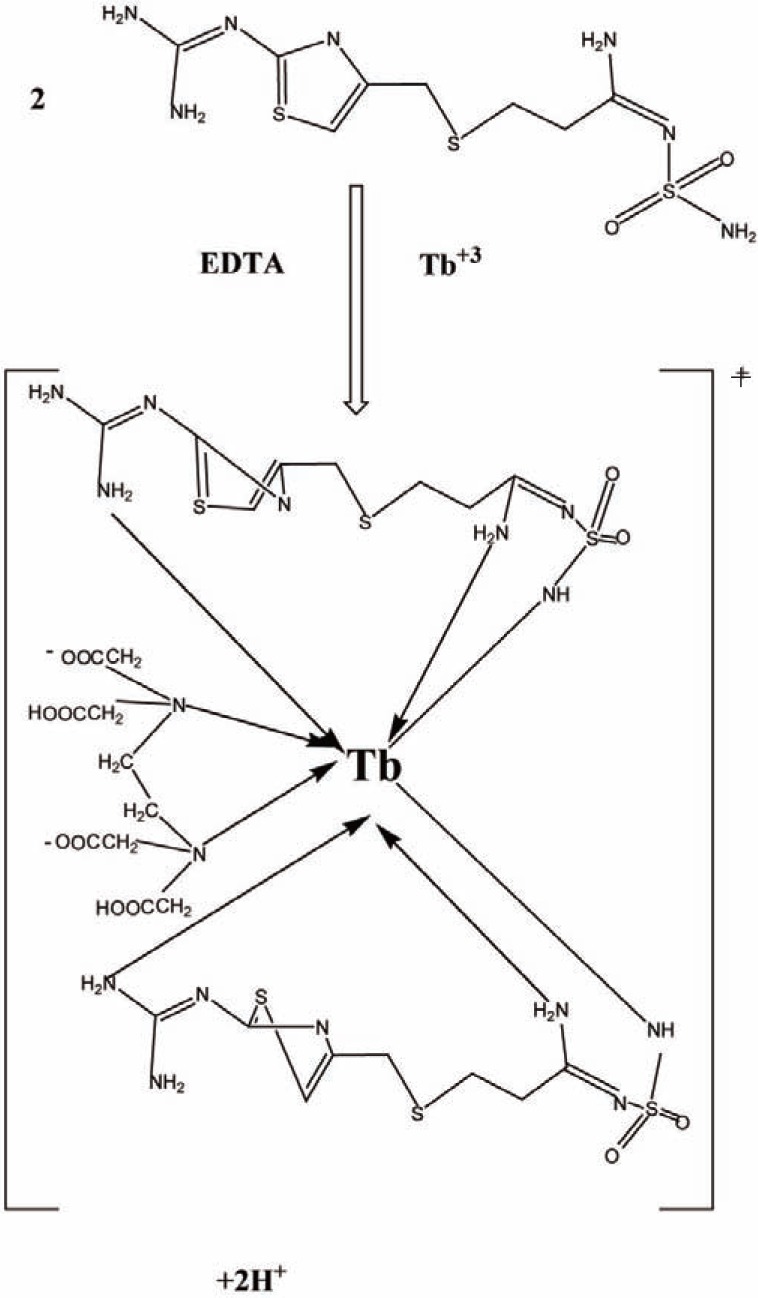
Proposal of the reaction pathway between famotidine and Tb^+3^ at pH4.0 in presence of EDTA.

The stoichiometry of the reaction between FMT with Tb^+3^, La^+3^ or Ce^+3^ was determined using Job’s continuous variation method (40) .For Job’s method, the plot reached a maximum value at a mole fraction of 0.7 for Tb^+3^ which indicated the formation of a 2:1 FMT - Tb^+3^, 0.5 for La^+3^ and Ce^+3^ presenting the formation of 1:1 FMT- La^+3^ or FMT- Ce^+3^(Fig. 4). The reaction pathway is proposed to proceed as shown in the Fig. 5 and Fig. 6.

### Pharmaceutical applications

The proposed method was further applied to the determination of FMT in its tablets and ampoules.

Common tablet excipients such as talc, lactose, starch, gelatin and magnesium stearate did not interfere with the assay. The results are abridged in Table 3.

**Table 3:** Application of the proposed and official methods for the determination of famotidine in its dosage forms

Pharmaceutical preparation	Amount taken (ng/ml)			Amount found (ng/ml)			found %			Official method (21)
	TbCl_3_	LaCl_3_	CeCl_3_	TbCl_3_	LaCl_3_	CeCl_3_	TbCl_3_	LaCl_3_	CeCl_3_	

Antodine®^a^ampoule (20famotidine/amp.)	20.0	10.0	10.0	20.05	10.13	9.97	100.25	101.30	99.70	101.23
	30.0	20.0	20.0	30.12	19.95	19.89	100.40	99.75	99.45	100.35
	40.0	30.0	30.0	39.84	30.25	30.32	99.60	100.83	101.06	100.75
	50.0	40.0	40.0	50.23	39.97	40.25	100.46	99.93	100.63	
	60.0	50.0	50.0	59.47	49.75	49.95	99.90	99.50	99.90	
	70.0	60.0	60.0	69.62	60.24	59.77	99.46	100.40	99.62	
X±SD							100.01 ± 0.36	100.29 ± 0.77	100.06 ± 0.67	100.78 ± 0.4
Student’s t test							0.41 (2.31)	0.57 (2.31)	0.79 (2.31)	
Variance ratio F test							1.49 (5.79)	3.06 (5.79)	2.32 (5.79)	
Antodine®^b^tablets (20 mg famotidine/tablet)	20.0	10.0	10.0	19.96	10.05	9.99	99.80	100.50	99.90	99.65
	30.0	20.0	20.0	30.09	20.14	20.21	100.30	100.70	101.05	99.44
	40.0	30.0	30.0	39.91	29.95	30.14	99.78	99.83	100.47	99.12
	50.0	40.0	40.0	50.14	39.78	39.93	100.28	99.45	99.83	
	60.0	50.0	50.0	59.85	49.88	50.06	99.75	99.76	100.12	
	70.0	60.0	60.0	69.66	60.08	60.17	99.51	100.13	100.28	
X±SD							99.90 ± 0.28	100.06 ± 0.53	100.28 ± 0.50	99.40 ± 0.27
Student’s t test							0.40 (2.31)	0.73 (2.31)	0.12 (2.31)	
Variance ratio F test							1.08 (5.79)	3.85 (5.79)	3.43 (5.79)	
Famotin®^c^tablets (40 mg famotidine/tablet	20.0	10.0	10.0	20.12	10.02	9.99	100.60	100.20	99.90	100.36
	30.0	20.0	20.0	30.06	19.91	19.88	100.20	99.55	99.40	100.48
	40.0	30.0	30.0	40.11	30.17	29.87	100.28	100.57	99.57	99.48
	50.0	40.0	40.0	49.99	39.96	40.06	99.98	99.90	100.15	
	60.0	50.0	50.0	60.22	49.88	50.14	100.37	99.76	100.28	
	70.0	60.0	60.0	69.93	60.09	59.84	99.90	100.15	99.73	
X±SD							100.22 ± 0.23	100.02 ± 0.39	99.84 ± 0.37	100.11 ± 0.55
Student’s t test							0.15 (2.31)	0.92 (2.31)	0.49 (2.31)	
Variance ratio F test							5.72 (5.79)	1.99 (5.79)	2.21 (5.79)	
Servipep®^d^tablets (40 mg famotidine/tablet	20.0	10.0	10.0	20.04	10.14	10.11	100.20	101.40	101.10	100.55
	30.0	20.0	20.0	30.14	20.14	19.92	100.47	100.70	99.60	100.65
	40.0	30.0	30.0	39.92	29.93	29.93	99.80	99.77	99.77	99.65
	50.0	40.0	40.0	49.89	39.85	49.93	99.78	99.63	99.86	
	60.0	50.0	50.0	59.92	49.97	50.06	99.87	99.94	100.12	
	70.0	60.0	60.0	70.14	60.08	59.99	100.20	100.13	99.98	
X±SD							100.05 ± 0.30	100.26 ± 0.75	100.07 ± 0.59	100.28 ± 0.55
Student’s t test							0.87 (2.31)	0.72 (2.31)	0.36 (2.31)	
Variance ratio F test							3.36 (5.79)	1.86 (5.79)	1.15 (5.79)	
Peptec®^e^ tablets (20 mg famotidine/tablet	20.0	10.0	10.0	19.98	9.98	10.06	99.90	99.80	100.60	100.45
	30.0	20.0	20.0	30.08	19.88	20.22	100.27	99.40	101.10	99.58
	40.0	30.0	30.0	39.73	30.12	29.96	99.32	100.40	99.87	99.65
	50.0	40.0	40.0	50.61	40.21	40.17	101.21	100.53	100.43	
	60.0	50.0	50.0	60.08	49.93	49.96	100.13	99.86	99.92	
	70.0	60.0	60.0	69.97	59.91	59.87	99.96	99.85	99.78	
X±SD							100.13 ± 0.62	99.97 ± 0.46	100.28 ± 0.51	99.89 ± 0.48
Student’s t test							0.56 (2.31)	0.93 (2.31)	0.50 (2.31)	
Variance ratio F test							1.67 (5.79)	1.09 (5.79)	1.13 (5.79)	

Figures between parentheses are the tabulated t and F values, respectively, at p=0.05 (38).

### Interferences

Many drugs which are frequently co- administered with FMT such as: magaldrate, dimethicone, and metformine were carefully tested. All of the studied compounds showed a negative interference as revealed by the low fluorescence intensity readings. The results are abridged in Table 4.

**Table 4:** Tolerance limits of co-administered drugs causing 3% relative error for a sample of famotidine 40 ng/ml

Drug	Fluorescence intensity			% Change in fluorescence			Tolerance limit (ng/ml)		
	Tb^+3^	La^+3^	Ce^+3^	Tb^+3^	La^+3^	Ce^+3^	Tb^+3^	La^+3^	Ce^+3^

Famotidine	442	502	199	-------	-------	-------			
Magaldrate	60	162	40	−86.43	−67.73	−79.89	7.24	17.21	10.72
Dimethicone	20	200	62	−95.48	−60.16	−68.84	2.41	21.25	16.62
Metformine	100	180	55	−77.38	−64.14	−72.36	12.07	19.12	14.74

### Stability study

**Figure 7:**
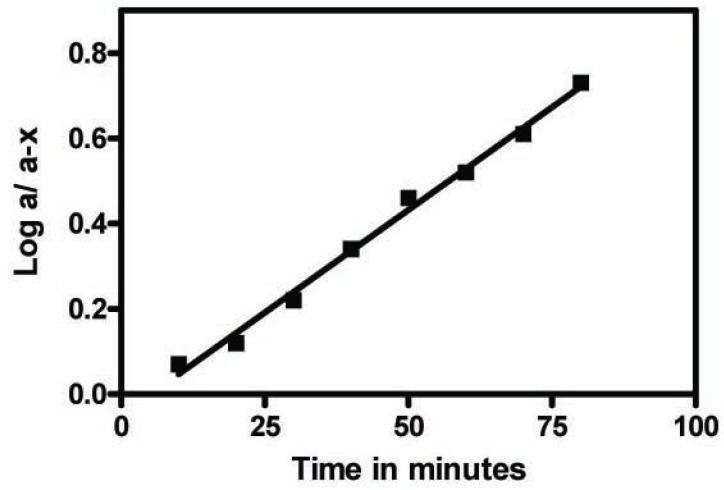
Semi log plot of FMT - Tb^+3^complex, FMT (40 ng/ml) versus different heating times (min) with 0.5M sodium hydroxide at 100°C.

**Figure 8:**
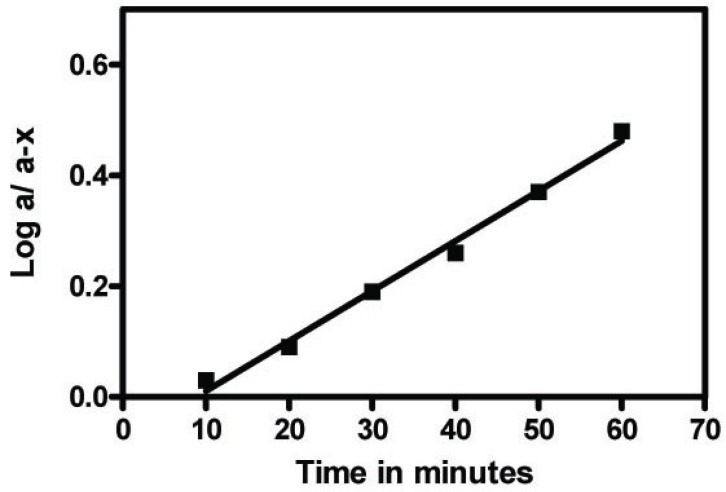
Semi log plot of FMT - Tb^+3^complex, FMT (40 ng/ml) versus different times (min) with 1% hydrogen peroxide on cold.

The proposed method is based mainly on the complex formation between lanthanide ions and an electron-donating partner. Degradation was attained upon induced alkaline treatment with sodium hydroxide (0.5 M) or oxidative degradation using hydrogen peroxide. Upon alkaline and oxidative degradation of FMT, the relative fluorescence readings of the fluorophore decreased gradually with time, thus indicating that the proposed method is a stability indicating one. The photo induced alkaline degradation of FMT followed first order kinetics (Fig. 7) with a rate constant K=0.012 min^-1^ and t_½_ was found to be 58 minutes using Tb^+3^. On the other hand, the oxidative degradation of FMT also followed first order kinetics (Fig. 8) with a rate constant K=0.008 min^-1^, and t_½_ was found to be 87 minutes using Tb^+3^, other metals behave similarly.

Complete alkaline or oxidative degradation was indicated from the disappearance of the fluorescence spectrum of the fluorophore. It was confirmed by performing a TLC scanning technique using a mobile phase consisting of chloroform: methanol: acetonitril (2:1:1) with UV detection, where the R_f_ values were found to be 0.81, 0.33, and 0.31 for famotidine, alkaline and oxidative degradation products respectively.

Complete alkaline degradation was attained after boiling with 2 M sodium hydroxide for one hour, while complete oxidative degradation was accomplished through the addition of 30% hydrogen peroxide and allowing the reaction mixture to stand for one hour at room temperature. The results of analysis of FMT intact drug in the presence of its degradation products is shown in Table 5. It is clear that the degradation products didn’t interfere with the assay of the intact drug.

Upon exposure of a methanolic solution of FMT to Deuterium lamp with a wavelength of 254 nm at a distance of 15 cm in a wooden cabinet for different time intervals (10 minutes interval up to 180 minutes), and then the proposed method was applied. It was found that only 25% of the drug was decomposed.

### Application to spiked and real human plasma

FMT is readily but incompletely absorbed from the gastrointestinal tract with peak concentrations in plasma occurring within 1 to 3 hours after oral dose (1). The serum therapeutic concentration range is 20–60 ng/ml (41), with a peak plasma level of about 156 ± 22 ng/ml reached within 3 hours (41). These value lie within the working concentration range of the proposed method, thus it could be successfully applied to the determination of FMT in both spiked and real human plasma over this range. The results are summarized in Table 5.

**Table 5:** Application of the proposed method for the determination of FMT in the presence of its alkaline and oxidative degradation products, and in spiked and real human plasma

Parameter	% found		
	Tb^+3^	La^+3^	Ce^+3^

Alkaline degradation FMT added = 20ng/ml			
X±SD	100.68 ± 0.87	100.18 ± 0.59	100.10 ± 0.58
Student’s t test	0.17 (2.78)	0.53 (2.78)	0.72 (2.78)
Variance ratio F test	1.97 (6.94)	1.10 (6.94)	1.14 (6.94)
Alkaline degradation FMT added= 40ng/ml			
X±SD	100.08 ± 0.84	99.99 ± 0.50	100.15 ± 0.46
Student’s t test	0.84 (2.78)	0.98 (2.78)	0.50 (2.78)
Variance ratio F test	1.84 (6.94)	1.54 (6.94)	1.82 (6.94)
Oxidative degradation FMT added = 20ng/ml			
X±SD	99.87 ± 0.71	100.88 ± 0.80	99.90 ± 0.67
Student’s t test	0.70 (2.78)	0.07 (2.78)	0.76 (2.78)
Variance ratio F test	1.31 (6.94)	1.66 (6.94)	1.17 (6.94)
Oxidative degradation FMT added = 40ng/ml			
X±SD	99.82 ± 0.48	99.89 ± 0.52	100.08 ± 0.73
Student’s t test	0.45 (2.78)	0.66 (2.78)	0.82 (2.78)
Variance ratio F test	1.67 (6.94)	1.42 (6.94)	1.39 (6.94)
Intra-day precision (spiked plasma)			
X±SD	100.28 ± 2.09	99.67 ±1.28	101.24 ± 1.50
Inter-day precision (spiked plasma)			
X±SD	100.39 ± 1.59	99.44 ± 1.73	100.30 ± 0.87
Intra-day precision (real plasma)			
X±SD	97.01 ± 0.75	103.21 ± 0.65	102.13 ± 0.74

Figures between parentheses are the tabulated t and F values, respectively, at p=0.05 (38).

### Precision

The within-day precision was evaluated through replicate analysis of plasma samples spiked with 50 ng/ml of the drug as cited in Table 6. The mean percentage recoveries based on the average of four separate determinations using either Tb^+3^ or La^+3^ and Ce^+3^ are summarized in Table 6.

**Table 6:** Validation of the proposed method for the determination of famotidine in biological fluids

Parameter	% found Repeatability (50 ng/ml)			%found Intermediate precision (40 ng/ml)		
	TbCl_3_	LaCl_3_	CeCl_3_	TbCl_3_	LaC1_3_	CeCl_3_

Spiked human plasma	98.89	98.89	98.88	100.89	100.43	100.75
	100.63	99.63	99.53	99.53	100.67	99.13
	99.55	98.55	99.15	99.25	98.85	99.85
	99.98	99.87	100.27	100.87	99.43	100.87
Mean found %	99.76	99.24	99.46	100.14	99.85	100.15
±SD	0.73	0.62	0.60	0.87	0.85	0.82
% RSD	0.73	0.62	0.60	0.87	0.85	0.82
	**% found Repeatability (150 ng/ml)**					
	**TbCl_3_**		**LaCl_3_**		**CeCl_3_**	

Real human plasma	98.56		100.32		99.32	
	99.12		99.02		100.69	
	100.95		98.23		100.05	
	99.77		99.54		98.12	
Mean found %	99.60		99.28		99.55	
±SD	1.03		1.35		1.67	
%RSD	1.03		1.35		1.67	

The inter-day precision was also evaluated through replicate analysis of plasma samples spiked with 40 ng/ml of drug on four successive days. The results are abridged in Table 6. On the other hand, the % recoveries of FMT in real human plasma were 99.60 ± 1.03, 99.28 ± 1.35 and 99.55 ± 1.67using Tb^+3^ or La^+3^ and Ce^+3^ respectively. The results are summarized in Table 6.

## DISCUSSION

The proposed method which depends on ternary complex formation of FMT with some lanthanide ions. It has the advantage over the previously reported methods as being more sensitive, simple, less time consuming and having lower detection limits.

The molecular structure of FMT is characterized by the presence of primary amine, sulphonamide and guanidine groups which are able to undergo complexation with lanthanides, and this initiated the present study.

Terbium chloride (TbCl_3_), lanthanum chloride (LaCl_3_), and cerous chloride (CeCl_3_) are members of the lanthanide group. Lanthanide ions emit weak fluorescence because of the weak absorption bands, and because they can be non-radiatively deactivated by the solvent molecules (35). The main advantages of lanthanide chelates in fluorescence spectrometry include large Stok's shift, narrow emission bands and long fluorescence life time (36).

Famotidine was found to form highly fluorescent complexes with TbCl_3_ at pH4, with LaCl_3_ at pH6.2, and with CeCl_3_ at pH7.2. The fluorophores were formed instantaneously and remained stable for more than 120 minutes.

## References

[1] Reynolds JEF (1999). Martindale. The Extra Pharmacopoeia.

[2] Apostu M, Bibire N, Dorneanu V (2005). UV spectrophotometric assay of famotidine in combination with picrolonic acid, picrolinate. Med. Chir. Soc. Med. Nat. Iasi.

[3] Rahman N, Kashif M (2003). Application of ninhydrin to spectrophotometric determination of famotidine in drug formulations. Farmaco.

[4] Rahman N, Kashif M (2003). Kinetic spectrophotometric determination of famotidine in commercial dosage forms. Anal. Sci.

[5] Ayad MM, Shalaby A, Abdellatef HE, Hosny MM (2003). New colorimetric methods for the determination of trazodone HCl, famotidine, and diltiazem HCl in their pharmaceutical dosage forms. Anal. Bioanal. Chem.

[6] Barańska M, Gumienna-Kontecka E, Kozlowski H, Proniewicz LM (2002). A study on the nickel II-famotidine complexes. J. Inorg. Biochem.

[7] Al-Ghannam S, Belal F (2002). Spectrophotometric determination of three anti-ulcer drugs through charge-transfer complexation. J. AOAC. Int.

[8] Chukwurah BK, Ajali U (2001). Quantitative determination of famotidine through charge-transfer complexation with chloranilic acid. Boll. Chim. Farm.

[9] Walash MI, Sharaf-EL-Din MK, Metwally ME, Shabana MR (2005). Kinetic spectrophotometric determination of famotidine in pharmaceutical preparations. J. Chin.Chem. Soc.

[10] Walash MI, Sharaf-EL-Din MK, Metwally ME, Shabana MR (2005). Polarographic determination of famotidine through complexation with Nickel (II) Chloride. J. Chin.Chem. Soc.

[11] Helali N, Monser L (2008). Stability indicating method for famotidine in pharmaceuticals using porous graphitic carbon column. J. Sep. Sci.

[12] Ashiru DA, Patel R, Basit AW (2007). Simple and universal HPLC-UV method to determine cimetidine, ranitidine, famotidine and nizatidine in urine: application to the analysis of ranitidine and its metabolites in human volunteers. J. Chromatogr. B.

[13] Tzanavaras PD, Verdoukas A, Balloma T (2006). Optimization and validation of a dissolution test for famotidine tablets using flow injection analysis. J. Pharm. Biomed. Anal.

[14] Zarghi A, Shafaati A, Foroutan SM, Khoddam A (2005). Development of a rapid HPLC method for determination of famotidine in human plasma using a monolithic column. J. Pharm. Biomed. Anal.

[15] Campanero MA, Bueno I, Arangoa MA, Escolar M (2001). Improved selectivity in detection of polar basic drugs by liquid chromatography-electrospray ionization mass spectrometry. Illustration using an assay method for the determination of famotidine in human plasma. J. Chromatogr. B.

[16] Zhong L, Eisenhandler R, Yeh KC (2001). Determination of famotidine in low-volume human plasma by normal-phase liquid chromatography/tandem mass spectrometry. J. Mass Spectrom.

[17] Simon RE, Walton LK, Liang Y, Denton MB (2001). Fluorescence quenching high-performance thin-layer chromatographic analysis utilizing a scientifically operated charge-coupled device detector. Analyst.

[18] Abdel Kader SA, Abdel Kawy MA, Nebsen M (1999). Spectrophotometric and spectrofluorimetric determination of famotidine and ranitidine using 1,4- benzoquinone. Anal. Lett.

[19] El- Bayoumi A, El- Shanawany AA, El-Sadek ME, Abd-El-Sattar A (1997). Synchronous spectrofluorimetric determination of famotidine, fluconazole and ketoconazole in bulk powder and in pharmaceutical dosage forms. Spectrosc.Lett.

[20] (2008). The British Pharmacopoeia.

[21] United States Pharmacopoeia XXX (2008). The National Formulary XXV.

[22] Fotopoulou MA, Ioannou PC (2002). Post-column terbium complexation and sensitized fluorescence detection for the determination of norepinephrine, epinephrine and dopamine using high-performance liquid chromatography. Anal. Chim. Acta.

[23] Egorova A, Beltyukova S, Teslyuk O, Karpinchik V (2001). Application of f--f luminescence of terbium ion for determination of non-steroidal anti-inflammatory drug-niflumic acid. J. Pharm. Biomed. Anal.

[24] Beltyukova SV, Tselik EI, Alla V, Egorova AV (1998). Use of sensitized luminescence of lanthanides in analysis of drugs. J. Pharm. Biomed. Anal.

[25] Miao YH, Hou FJ, Jiang CQ (2005). Determination of heparin using ciprofloxacin-Tb^3+^ as a fluorescence probe. Anal. Sci.

[26] Wei W, Wang H, Jiang C (2006). Spectrofluorimetric determination of trace heparin using lomefloxacin-terbium probe. Spectrochem. Acta Part A.

[27] Rodríguez-Díaz RC, Fernandez-Romero JM, Aguilar-Caballos MP, Gómez-Hens A (2006). Determination of fluoroquinolones in milk samples by postcolumn derivatization liquid chromatography with luminescence detection. J. Agric. Food Chem.

[28] Ocana JA, Callejon M, Barragan FJ (2000). Terbium-sensitized luminescence determination of levofloxacin in tablets and human urine and serum. Analyst.

[29] Ocana JA, Callejon M, Jose-Barragan F (2001). Application of terbium-sensitized luminescence for the determination of grepafloxacin in human urine and serum. J. Pharm. Sci.

[30] Chen S, Ma HW, Zhao HC, Feng RQ (2004). Terbium-sensitized fluorescence method for the determination of pazufloxacin mesilate and its application. Anal. Sci.

[31] Zhang X, Ouyang J, Zhai S, Huang G (2005). Flow-injection with enhanced chemiluminescence detection of ofloxacin in human plasma. Luminescence.

[32] Nie LH, Zhao HC, Wang X, Yi L (2002). Determination of lomefloxacin by terbium sensitized chemiluminescence method. Anal. Bioanal. Chem.

[33] To CL, Xiang GH, Liu WP (2005). Study on the determination of ciprofloxacin by the terbium (III) ion fluorescence probe sensitized by the surfactant. Guang Pu Xue Yu Guang Pu Fen Xi.

[34] Karim MM, Lee SH (2008). Determination of Enoxacin Using Tb Composite Nanoparticles Sensitized Luminescence Method. J. Fluoresc.

[35] George J (1993). Lanthanide- sensitized luminescence and applications to the determination of organic analytes. Analyst.

[36] Diamandis EP, Christopoulos TK (1990). Europium and terbium chelators for time resolved fluorometric assays. Anal.Chem.

[37] Guidance for industry; Q2B of analytical procedure: Methodology. http://www.fda.gov/eder/guidance/1320fnl.

[38] Miller JC, Miller JN (2005). Statistics for Analytical Chemistry.

[39] Inczedy J (1976). Analytical and Application of Complex Equilibria.

[40] Sawyer DT, Heineman WR, Beebe JM (1984). Chemistry Experiments for Instrumental Methods.

[41] Moffat AC, Osselton MD, Widdop B (2004). Clarke's Analysis of Drugs and Poisons.

